# Improving cancer patient care: development of a generic cancer consumer quality index questionnaire for cancer patients

**DOI:** 10.1186/1471-2407-13-203

**Published:** 2013-04-23

**Authors:** Judith C Booij, Marieke Zegers, Pauline MPJ Evers, Michelle Hendriks, Diana MJ Delnoij, Jany JDJM Rademakers

**Affiliations:** 1 Department of Demand-Driven Healthcare, Netherlands Institute for Health Services Research (NIVEL), PO box 1568, Utrecht, 3500 BN, The Netherlands; 2 Dutch Federation of Cancer Patient Organisations (NFK), PO box 8152, Utrecht, 3503 RD, The Netherlands; 3 Centre for Consumer Experience in Healthcare (Centrum Klantervaring Zorg, CKZ), Utrecht, The Netherlands/Tranzo (Scientific Centre for Transformation in Health and Social Care), Tilburg University, PO box 1568, Utrecht, 3500 BN, The Netherlands

**Keywords:** Consumer Quality Index (CQI), Focus groups, Healthcare evaluation, Healthcare quality, Patient experience, Quality indicators

## Abstract

**Background:**

To develop a Consumer Quality Index (CQI) Cancer Care questionnaire for measuring experiences with hospital care of patients with different types of cancer.

**Methods:**

We derived quality aspects from focus group discussions, existing questionnaires and literature. We developed an experience questionnaire and sent it to 1,498 Dutch cancer patients. Another questionnaire measuring the importance of the quality aspects was sent to 600 cancer patients. Data were psychometrically analysed.

**Results:**

The response to the experience questionnaire was 50 percent. Psychometric analysis revealed 12 reliable scales. Patients rated rapid and adequate referral, rapid start of the treatment after diagnosis, enough information and confidence in the healthcare professionals as most important themes. Hospitals received high scores for skills and cooperation of healthcare professionals and a patient-centered approach by doctors; and low scores for psychosocial guidance and information at completion of the treatment.

**Conclusions:**

The CQI Cancer Care questionnaire is a valuable tool for the evaluation of the quality of cancer care from the patient’s perspective. Large scale implementation is necessary to determine the discriminatory powers of the questionnaire and may enable healthcare providers to improve the quality of cancer care. Preliminary results indicate that hospitals could improve their psychosocial guidance and information provision.

## Background

Cancer patients have to cope with a great deal of distress. A recent study among patients with inoperable lung cancer showed that for 27 percent of these patients their experiences with healthcare services were among their most important concerns. Waiting times, problems with information and communication and a lack of continuity in healthcare professionals are among the healthcare experiences that cause distress [1]. In 2001 the Institute of Medicine (IOM) postulated the theory of patient-centeredness as one of the ways in which the healthcare system could reduce patients’ problems, instead of add to their burden. The IOM states that care should be patient-centered, that it is respectful of and responsive to individual patient’s preferences, needs, and values and that patient values should guide all clinical decisions [2]. Patient-centered care has been associated with improved patient satisfaction, better treatment adherence, improved recovery and health outcomes, reduced readmission rates and better seeking of follow-up care [3-6]. Therefore, patient-centeredness of care is now seen as an important quality of care aspect worldwide. In order to achieve patient centered care measuring patient experiences with healthcare is vital. Comparative measures of patient preferences can be used by healthcare professionals to improve care, by patients to select their caregiver, by insurers to contract doctors and hospitals and by hospital managers and policy makers to monitor the quality of care [2,7].

It is important to note that healthcare professionals and patients do not always agree on what is important in patient care. In 2010, Wessels et al. reported that expertise and attitude of healthcare providers as well as accessibility of services were more important to cancer patients than healthcare professionals expected. Moreover, the importance of organisational and environmental aspects was overrated by healthcare professionals [8]. Patient centeredness can only be measured by asking the patients themselves. When measuring patient experiences, elements of patient centeredness as identified in the literature (respect for patient needs and preferences, involvement of family and friends, sensitivity to nonmedical and spiritual dimensions of care, collaboration and team management, education and shared knowledge, free flow and accessibility of information) must be included [9].

For measuring patient experiences, valid and reliable instruments that have been developed in close interaction with patients should be used. In the Netherlands, patients’ experiences are measured using a standardized method, the Consumer Quality Index (CQI) [10]. This CQI consist of a large ‘family’ of questionnaires and interview protocols that are complemented with detailed instructions about how to use these instruments (e.g. how to draw samples, how to collect data, how to analyse and report findings etc.). The CQI has been implemented in numerous areas of healthcare where it provides information for care providers to improve their service, for policy makers to aid in determining policy, for health care insurers to use in their negotiations with healthcare organisations and for patients to help them make informed choices between healthcare providers [11]. Although literature is inconsistent, Fung et al. show in their review that the availability of publically accessible performance data stimulates quality improvement at the hospital level[12-14]. The CQI methodology is based on the American CAHPS (Consumer Assessment of Healthcare Providers and Systems) [15] and the Dutch QUOTE (QUality of care through the patient’s eyes) [16] instruments. It entails a unique combination of questions on the frequency with which quality criteria are met and the importance of aspects according to patients.

Cancer patients are generally regarded to have different needs than patients who have not been confronted with a potentially lethal disease. A number of studies has measured the satisfaction and needs of cancer patients with their healthcare [17-19]. Patients rated technical quality of medical care, interpersonal and communication skills of doctors and accessibility of care as important aspects [17]. Skarstein reported in 2002 that the most important predictors of cancer patient satisfaction were performance of nurses and physicians, level of information perceived, outcome of health status, reception at the hospital and anxiety [18]. In 2003, Tamburini reported information needs (regarding diagnosis, future conditions, dialogue with doctors, economic insurance solutions related to the disease) and improved ‘hotel’ services (bathrooms, meals, cleanliness) as important aspects [19]. In 2009, a questionnaire measuring cancer patients’ preferences was developed, based on the outcome of 10 focus group interviews. Patients rated expertise, safety, performance and attitude of physicians and nurses as the most important issues in cancer care [20].

Although it is important to measure the needs of patients, their satisfaction and preferences, as was done in the above-mentioned studies, it is more informative to combine this with measuring patients’ actual experiences with healthcare [16]. Reports of experiences rather than satisfaction can give more direct guidance on how to improve the provided healthcare [21]. In the Netherlands, a questionnaire on patient experiences has been developed for breast cancer patients [10]. However, until now a generic questionnaire capable of measuring experiences of patients with all types of cancer was not available. A generic questionnaire has advantages: it can be used for patients with all tumour types and makes developing many tumour-specific questionnaires superfluous. Moreover, a generic questionnaire can be set out among large patient groups, thereby making it easier to benchmark quality of cancer care on a hospital level.

We assumed that from the patient’s perspective, the majority of healthcare experiences are not tumour-specific. Therefore, the aim of the current study was to develop a generic questionnaire to measure both preferences and experiences of patients with all types of cancer. The questionnaire should be suitable for registration and improvement of quality of cancer healthcare, through, for instance, the implementation in the auditing procedure of hospitals. For the development of the questionnaire we combined multiple sources and worked in close collaboration with the Dutch Federation of Cancer patient Organisations (NFK) in order to ensure robust and relevant outcomes that can be implemented in the cancer care system. We performed focus group interviews with cancer patients, looked at quality criteria devised by the NFK and already existing questionnaires. The developed questionnaire was tested in a group of cancer patients.

In addition to our question, can we develop a questionnaire that measures the actual experiences of cancer patients, our research questions were:

1. What are the most important aspects of quality of care according to cancer patients?

2. What are the actual experiences with these quality aspects?

3. Are there any differences in experiences between subgroups of cancer patients?

## Methods

A group of experts was formed, consisting of healthcare professionals, patient organisations, health insurers and researchers. This group was consulted for decisions regarding the creation and adaptation of the questionnaire.

Patients with all types of malignant tumours were included, as long as they received treatment in a Dutch hospital or specialized cancer treatment centre. We chose the following inclusion criteria for both the focus group discussions and the questionnaires. Participants were at least 18 years old and the last treatment occurred no longer than two years before the focus group discussion or conduct of the survey. This study was performed in agreement with the declaration of Helsinki. Approval by a medical ethics committee was not required. All participants in this study gave written informed consent for the use of the data provided by them. Data from focus group discussions and questionnaires were analyzed anonymously.

### Sampling

The chairmen of the cancer patient organisations affiliated to the Netherlands Federation of Cancer patient organisations (NFK) invited all their active members, ever diagnosed with cancer, through letters or e-mails to participate in the focus group discussions.

The claims data of a Dutch healthcare insurance company with national coverage, were used to draw a random selection of 1,489 patients, ever diagnosed with cancer, who received cancer care in any hospital in the Netherlands, or in a specialized cancer center in the last two year for the experience questionnaire. Patients were selected using diagnosis-related groups codes (DRG codes). It concerned patients with the following types of cancer: lung, breast, colorectal, prostate, haematological, gynaecological and skin. Basal cell cancer patients were excluded (on their DRG code), since these patients are not always told they have cancer. Receiving a questionnaire concerning cancer would cause unnecessary distress. Patients in all phases of their treatment were included, receiving surgical treatment, chemotherapy, radiotherapy, immune therapy or hormonal treatment, with the exception of patients in a palliative phase, who were excluded through their DRG code. A second random sample of 600 patients using the same criteria was drawn for the importance questionnaire.

### Construction of the questionnaire

Three focus group discussions were held with seven, nine and nine cancer patients in November and December of 2009. Each focus group discussion was chaired by a researcher from the Centre for Consumer Experience in Healthcare (Centrum Klantervaring Zorg, CKZ) and Netherlands Institute for Health Services Research (NIVEL) and a policy officer from the NFK. Discussions were audio-taped with permission of the participants. Each participant was asked to write down three positive and three negative experiences with the hospital care surrounding their referral, diagnosis, treatment or aftercare. Subjects were then discussed in the group. Two researchers independently analyzed transcripts from the focus group discussions for the presence of quality aspects using descriptive thematic analysis.

Relevant items mentioned more than once were included in the questionnaire, along with additional important items from the following sources. These included a list of quality aspects for cancer care from the patient’s perspective created by the NFK [22] and a list of general quality criteria from the patient perspective in healthcare constructed by the Netherlands patient consumer federation (NPCF) [23]. We also included quality aspects from three questionnaires. The first was the EORTC-IN-PATSAT32 questionnaire from the European Organisation for Research and Treatment of Cancer (EORTC). This questionnaire is used to assess in-patient satisfaction with cancer care [24]. The second questionnaire was the CQI breast care [10] developed by the NIVEL and CKZ to assess patients’ experiences with breast care. The third was the CQI hospital care [25], developed by the NIVEL and CKZ, that measures the quality of hospital care.

We developed an ‘experience’ questionnaire that measures the experiences of patients and an ‘importance’ questionnaire to assess the importance patients attach to each quality aspect. Both questionnaires contained a number of questions regarding patient characteristics. All other questions in the experience questionnaire had one of the following response categories ‘never-sometimes-usually-always’, ‘no not at all-somewhat-largely-yes completely’, ‘none-some-most-all’, or one through ten for grades. Responses, with the exception of grades, were converted into a scale of one to four, where the highest score was the most positive answer. The importance questionnaire included all experience questions from the experience questionnaire, with the response categories ‘not important-somewhat important-quite important-very important’. These responses were also converted into a scale of one to four, four being very important.

### Pre-testing

All patients who took part in the focus group discussions received the experience questionnaire with instructions to comment on the clarity of the questions. They were asked to judge how long it took them to fill out the questionnaire, to give comments on unclear wording, to record if questions were clear and if all important aspects were covered in the questionnaire. The questionnaire was filled out by 20 cancer patients. Unclear or incorrect questions were altered before data collection.

### Data collection

We based our data collection on the Dillman method[26]. In April 2010 (week 0) an invitation letter with a link to the online ‘experience’ questionnaire was sent to 1,498 cancer patients. An invitation with a link to the ‘importance’ questionnaire was sent to 600 additional patients. In week 1, a note was sent thanking respondents and reminding non-responders to a link to the questionnaire. In week 4 another reminder was sent to the non-responders along with a paper version of the questionnaire. Finally, in week 6, a note was sent, thanking respondents and reminding non-responders to the questionnaire through a link to the website.

### Analyses

Data were analyzed following the CKZ manual on the development of CQI questionnaires [27]. In short, the data entry of ten percent of the paper questionnaires was checked for errors. Up to one percent was allowed. Subsequently, a histogram was made for all questions to identify values that are outside the scope of the answer categories. Double entries (on patient identification number), and non-responders (with and without statement of a reason) were removed from the dataset. Questionnaires that were not filled out by the patient the questionnaire was sent to, were removed. Finally, questionnaires where less than 50 percent of mandatory questions were answered (<41) were removed. All questions were considered mandatory with the exception of questions following a ‘skip-question’, a question where the answer given may redirect the respondent to a certain question, thereby skipping questions that are irrelevant. Representativeness of respondents was checked by comparing our data on age, gender and tumour type with numbers from the Dutch National cancer registration (NKR) [28].

### Importance questionnaire

For the importance questions we calculated the percentage of responses in the highest category (very important). We regarded the top 10 questions to be the most important questions in the questionnaire and the bottom 10 questions to be eligible for removal from the questionnaire.

### Experience questionnaire

For the experience questionnaire we performed analyses on item-level to identify extremely skewed items (as a rule of thumb: more than 90 percent of responses in the most positive or the most negative answer category) and questions with a high non-response (as a rule of thumb: more than 5 percent). Subsequently, we performed inter-item analyses to identify questions with a large overlap in response. If two questions had a Pearson’s correlation coefficient exceeding 0.70 and overlap concerning content, one of the questions was considered potentially redundant.

Prior to carrying out factor analyses on the experience questionnaire, we removed extremely skewed items and redundant questions. All remaining 73 questions with a 1 to 4 answer-scale were included in the factor analysis. We performed principal component analysis with oblique rotation in order to ascertain the underlying structure of the questionnaire while taking into account a certain minimal amount of overlap between factors due to the fact that people who respond positively to certain questions may also respond positively to other questions. Questions with a factor load of more than 0.3 for a certain scale were included in the scale where they had the highest factor load.

We performed reliability analyses on the formed scales. The reliability of each scale was measured with Cronbach’s α. A scale with an α greater than 0.70 was considered reliable, a scale with an α between 0.60 and 0.70 was considered to be moderately reliable and was not removed from the questionnaire. However, a moderately reliable scale needs to be re-evaluated in future measurements. The correlation of a question to all questions in its scale (item-test correlation (ITC)) was required to be greater than 0.40 for inclusion in the scale. We also registered those questions for which removal of the question from the scale would lead to an increase in the reliability of the scale (Cronbach’s α).

Subsequently, we calculated average scale scores with 95% confidence intervals (95% CIs) for all the scales in the experience questionnaire and compared the averages for the five most frequently reported tumour types using ANOVA.

We calculated improvement scores in order to identify quality aspects where patients had negative experiences while regarding the aspect as important. These improvement scores combine data from the experience questionnaire and the importance questionnaire. Scores are defined by the mean score of a question (importance questionnaire) times the percentage of people with a negative experience (experience questionnaire) times 100 [10,16].

We discussed all results with the group of experts. The final decision for removal of questions from a scale and from the questionnaire was based on the results from the psychometric analyses and on arguments from the group of experts with respect to the content of the questions.

For international use, the final questionnaire was translated, reviewed by a panel of experts and back-translated according to the WHO guidelines [29].

## Results

### Focus groups

In total 33 people registered for a focus group discussion, 24 ultimately attended. The 24 participants were distributed over the three focus group discussions, 33 percent were male and 67 percent were female. Participants had a mean age of 57 years (sd=8, range 25–86). Participants had tumours in one or more of 11 different tissues or organs, the top three most frequently reported included blood and bone marrow, breast and digestive tract. Analysis of the focus group discussions together with the quality aspects derived from the quality lists of the NFK and the NPCF, the CQI questionnaires on breast cancer care and hospital care and the EORTC-IN-PATSAT32 questionnaire resulted in 19 quality aspects. These included hospital related topics (patient-centered approach, waiting times, information, communication, skills of health professionals, psychosocial care, cooperation between professionals, provisions in and accessibility of the hospital, after care) and personal topics (autonomy, safety, heritability, pain, fatigue, concentration difficulties, sexuality, nutrition and medication); full list available on request.

### Pre-testing and experience questionnaire

The experience questionnaire used for pre-testing contained 110 questions. There were 8 questions to determine the type of cancer and hospital care received and 14 questions on demographic characteristics of the participants (termed ‘About yourself’). Six questions, so called skip-questions, were designed to direct participants through the questionnaire. Some of these skip-questions also measured patient experiences and were therefore included in the analyses. The remaining 82 questions measured patient experiences.

The questions in the experience questionnaire were grouped into the following themes: organisation of the hospital, patient-centered approach by nurses, patient-centered approach by doctors, information and communication, personal input, skills and knowledge of the health professionals, cooperation and communication between healthcare professionals, guidance and support, end of the treatment, after care and grading of the hospital. For pre-testing the questionnaire was filled out by twenty cancer patients. Their input was used to adjust the questionnaire, wording was clarified and answer categories were adjusted. No questions were removed. The adjusted version of the questionnaire was subsequently used.

### Importance questionnaire

The ‘importance’ questionnaire contained the same questions as the ‘experience’ questionnaire on type of cancer, hospital care and demographics. The remaining 80 questions corresponded to 80 of the 83 experience questions in the pilot questionnaire. Three questions regarding grading of hospital and healthcare professionals were not included in the ‘importance’ questionnaire.

### Response

Table 1 reports background characteristics of the respondents. Tumour type indicates the tumour type reported by the patient, not the tumour type from the sample drawn. Several tumour types were reported in addition to the ones from the inclusion criteria since the questionnaire is designed for use in all tumour types. Respondents could indicate more than one tumour type.

**Table 1:** Background characteristics of the focus group participants, the respondents to the importance questionnaire and the respondents to the experience questionnaire

	**Focus group**		**Importance questionnaire**		**Experience questionaire**	
	**N**	**%**	**N**	**%**	**N**	**%**
**Age (years):**						
18-24	0	0	3	0.9	2	0.2
25-34	1	4.2	1	0.3	9	1.2
35-44	1	4.2	13	4.1	32	4.4
45-54	9	37.5	38	11.8	89	12.3
55-64	9	37.5	81	25.2	204	28.1
65-74	3	12.5	113	35.2	227	31.3
75 or older	1	4.2	72	22.4	163	22.5
**Gender:**						
male	8	33.3	167	52.0	333	46.1
female	16	66.7	154	48.0	389	53.9
**Highest education:**						
none	0	0	7	2.3	11	1.6
Primary education (1)	0	0	26	8.5	59	8.4
Lower secondary education (2)	1	4.2	63	20.7	158	22.4
Upper secondary education (3)	1	4.2	64	21.0	167	23.7
Post-secondary non-tertiary education (4)	3	12.5	37	12.1	85	12.1
Short cycle tertiary education (5)	5	20.8	27	8.9	69	9.8
Bachelor (6)	7	29.2	59	19.3	111	15.8
Master/Doctoral (7 or higher)	7	29.2	21	6.9	32	4.6
**Experienced health:**						
excellent	2	11.1	18	5.7	46	6.4
very good	3	16.7	57	18.0	102	14.2
good	6	33.3	172	54.3	367	51.0
reasonable	7	38.9	54	17.0	169	23.47
poor	0	0	16	5.1	36	5.0
**Tumour type** (more than 1 answer possible)			total 408		total 732	
Digestive tract	6	17.1	58	14.2	205	28
Lower respiratory tract	3	8.6	61	15.0	67	9.2
Breast	9	25.7	45	11.0	171	23.3
Male reproductive organs	1	2.9	79	19.4	153	20.9
Skin	1	2.9	43	10.5	70	9.6
Blood, bone marrow and lymph nodes	7	20.0	29	7.1	45	6.1
Urinary tract	2	5.7	13	3.2	9	1.2
Female reproductive organs	3	8.6	16	3.9	73	10.0
Head and neck	1	2.9	16	3.9	4	0.5
Central nervous system			0	0	2	0.3
Bone or soft tissue			4	1.0	12	1.6
Endocrine glands	1	2.9	3	0.7	4	0.5
Eye and orbit	1	2.9	2	0.5	3	0.4
**Treatment received** (more than 1 answer possible)						
surgery	21	45.7	230	44.6	492	46.9
chemotherapy	11	23.9	115	22.3	141	13.4
radiotherapy	12	26.1	125	24.2	257	24.5
hormone treatment	2	4.3	44	8.5	127	12.1
immune therapy			2	0.4	32	3.1
**Treating specialist**						
surgeon	13	19.7				
medical specialist/oncologist	13	19.7				
radiologist/radiotherapist	8	12.1				
oncology nurse	5	7.6				
nurse practitioner	3	4.5				
psychologist/social worker	6	9.1				
general practitioner	12	18.2				
physical therapist	2	3.0				
dietitian	3	4.5				
dermatologist	1	1.5				
**Stage of treatment**						
tests to acertain diagnosis			4	1.3	13	1.9
diagnosis known, treatment starts			4	1.3	8	1.2
during treatment			68	22.2	158	22.9
no further treatment possible			7	2.3	14	2.0
non-curative treatment			31	10.1	69	10.0
checks after treatment			168	54.9	374	54.1
treatment and checks after treatment are finished			15	4.9	42	6.1
I don’t know (anymore)			9	2.9	13	1.9

Educational levels were described using the International Standard Classification of Education (ISCED) categories (one through seven).

The experience questionnaire was sent to 1,498 patients. This sample was representative for the Dutch cancer patient population concerning age and gender. The people in the sample for the experience questionnaire were only slightly older, 6 percent was below 45 years, compared to 9 percent in the cancer patient population; 34 percent was above 75 years, compared to 30 percent in the cancer patient population. In the sample 49 percent were male, compared to 52 percent in the cancer patient population. The questionnaire was completed by 732 participants (50 percent), 46 percent was male, 54 percent was female. The mean age of participants was 65.8 years (se 0.45 years), see Table 1. Participants reported a malignancy in one or more of 14 different tissues or organs (see Table 1), the three most common were: digestive tract (28 percent), breast (23 percent) and male reproductive organs (21 percent).

The sample for the importance questionnaire also contained 6 percent under 45 years and 34 percent above 75 years, slightly older than the population of cancer patients (Table 2). Furthermore, 48 percent was male, compared to 52 percent in the cancer patient population. The importance questionnaire was filled out by 408 participants (68 percent). The age distribution in this group was comparable to that of the experience questionnaire (mean 67.1 years, se 0.56 years), 52 percent was male and 48 percent was female. The three most common tumours in this group were cancer in the male reproductive organs (19 percent), lung cancer (15 percent) and cancer in the digestive tract (14 percent) (Table 1).

**Table 2:** **Background characteristics of the Dutch cancer patient population **[28]

	**Dutch cancer patient population (2007)**
**Age (years)**	**%**
<45	9
45-60	21
60-75	40
>75	30
**Gender**	
male	52
**Tumour type**	
Digestive tract	19
Lower respiratory tract	13
Breast	20
Male reproductive organs	16
Skin	14
Blood, bone marrow and lymph nodes	10
Urinary tract	8
Female reproductive organs	6
head and neck	4.2
central nervous system	1
bone or soft tissue	2.6

### Important aspects

The ten most and least important aspects, based on the results from the importance questionnaire, can be found in Table 3. No significant differences were observed in importance for the five most common tumour types using ANOVA analyses (data not shown). Patients rated rapid and adequate referral, consultation of other doctors by the doctor if additional expertise is required, rapid start of the treatment after diagnosis, enough information and confidence in the health professionals as the most important themes in the questionnaire.

**Table 3:** The ten most and least important quality aspects according to cancer patients (importance questionnaire)

**Rank**	**Do you find it important that:**	% very important*	**Average importance score # (95% CI)**
**Most important questions**			
1	your family doctor refers you to hospital as quickly as you would like	75	3.72 (3.67-3.78)
2	your treatment is started as soon after the diagnosis as you would like	74	3.72 (3.67-3.78)
3	there are regular checks to find new tumours, if your type of cancer is heritable	68	3.63 (3.56-3.70)
4	your doctor consults other doctors or refers you if additional expertise is required	68	3.65 (3.59-3.71)
5	the time between first examination and results was short	66	3.62 (3.55-3.68)
6	the time between referral by the family doctor and the first examination was less than 6 days	65	3.59 (3.52-3.66)
7	you have confidence in the doctors and nurses in the hospital	65	3.63 (3.57-3.68)
8	doctors and nurses in the hospital give you the best possible care	61	3.60 (3.54-3.66)
9	you regularly receive information about the effect of the treatment, during the treatment period	57	3.54 (3.48-3.60)
10	you receive sufficient information about the (dis-) advantages of different treatments, so you can make a proper choice	56	3.50 (3.43-3.57)
**Least important questions**			
1	careproviders inform you about patient organisations	10	2.53 (2.44-2.63)
2	you can talk to doctors and/or nurses about alternative treatments and medicines, if you wish	15	2.66 (2.56-2.76)
3	you are informed if the waiting time is longer than expected	17	2.80 (2.71-2.89)
4	nurses are polite to you	18	2.98 (2.91-3.06)
5	nurses show personal interest in you	18	2.89 (2.80-2.97)
6	you are offered help with questions regarding resuming you regular activities, during aftercare	19	2.76 (2.65-2.86)
7	you receive information from the hospital about possibilities for psychosocial guidance, during aftercare	19	2.86 (2.77-2.95)
8	it is regularly checked if you need help dealing with the emotions brought about by the disease and treatment	19	2.81 (2.72-2.91)
9	you are called at home by a care provider from the hospital within a week after completion of the treatment, to discuss how you are doing	19	2.81 (2.71-2.90)
10	the waiting time for outpatient appointments in the hospital is less than 15 minutes	20	2.78 (2.68-2.87)

Questions were translated from Dutch for this article. * The percentage of people who scored the item as being very important. # Importance scores range from 1 (not important) to 4 (very important).

### Psychometric analyses

The psychometric analyses were performed on all questions from the experience questionnaire measuring patient experiences. There were no questions extremely skewed, or with more than 5 percent non-response. We identified five pairs of questions with a high mutual correlation and overlapping content. Of the 83 experience questions, 55 fit into 11 reliable scales and one moderately reliable scale. Each scale contained between three and six questions. The scales contained questions regarding the different time frames: treatment, after care and referral to other healthcare professionals. Within these time frames, there were scales regarding personal attention, freedom of choice, guidance by and skills of healthcare professionals, communication and the patient centered approach of both doctors and nurses. The scales and corresponding example questions can be found in Table 4. The table contains the twelve scales identified in the questionnaire before and after adjustment of the scales based on the psychometric analyses. Before adjustment eleven scales were reliable, one was moderately reliable. After adjustment all twelve scales were reliable.

**Table 4:** Reliability of the scales in the experience questionnaire, before and after removal of redundant questions based on psychometric analyses

	**Scale**	**Example of question**	**Before adjustment**		**After adjustment**	
			**no. questions**	**α**	**no. questions**	**α**
1	Personal attention during aftercare	During aftercare, was attention paid to complaints of fatigue?	6	0.80	5	0.77
2	Cooperation and communication between healthcare professionals	Was the advice given by different healthcare professionals congruent?	4	0.78	3	0.81
3	Freedom of choice	Were you given enough time to choose a treatment?	3	0.79		
4	Skills and cooperation of healthcare professionals	Did doctors and nurses in the hospital give you the best possible care?	6	0.76		
5	Psychosocial guidance	Did the hospital provide you with information about guidance for dealing with emotions and practical issues caused by cancer?	5	0.83	4	0.86
6	Other investigations and treatments	Was it possible to discuss a second opinion with your doctor?	4	0.76		
7	Information during treatment	Did the healthcare professionals give you enough information?	6	0.79		
8	Continuity of care by healthcare professional/side effects and complaints	Were doctors and nurses prepared to talk to you about things you thought had gone wrong?	4	0.71		
9	Patient-centered approach by doctors	Did the doctors listen carefully to you?	5	0.87	4	0.86
10	Patient-centered approach by nurses	Did the nurses take you seriously?	5	0.86	4	0.85
11	Information at completion of treatment	At completion of the treatment, did you receive information about the possibility of psychosocial care?	4	0.68	3	0.73
12	Transfer to other healthcare professionals	Were important persons and institutions (general practitioner, rehabilitation) informed of the completion of your treatment?	3	0.75		

no. questions: the number of questions each scale contains. α: Cronbach’s alpha,. Scales for which the fields ‘after adjustment’ are blank, were not altered.

### Adjustment of the questionnaire

Eleven questions were eligible for removal from the questionnaire because they reduced the reliability of a scale, were not considered important by respondents, had high inter-item correlation, or a combination. The group of experts made the final decision for removal of 10 items from the questionnaire and merger of 2 questions into one. This resulted in a questionnaire containing 99 questions, see additional file 1. Six of the removed questions were part of a scale, for three of these, removal increased the alpha value of the scale, for the other three removal decreased the α. The adjusted scales all had an α value greater than 0.70 and were therefore reliable, see Table 4. The English translated version of the adjusted questionnaire can be found in Additional file 1.

### Scale scores

**Figure 1:**
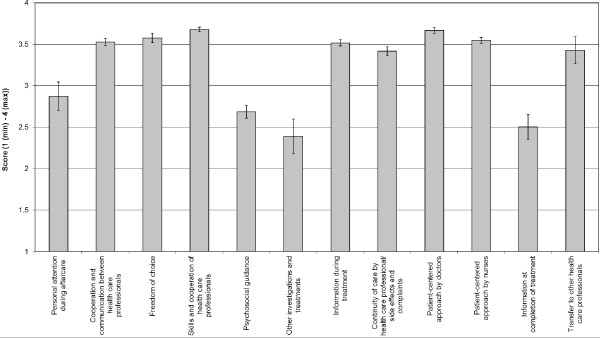
**Average scale score and 95% confidence intervals for the twelve scales of the experience questionnaire.** Scores ranged from 1 to 4, 4 being the best possible score.

**Figure 2:**
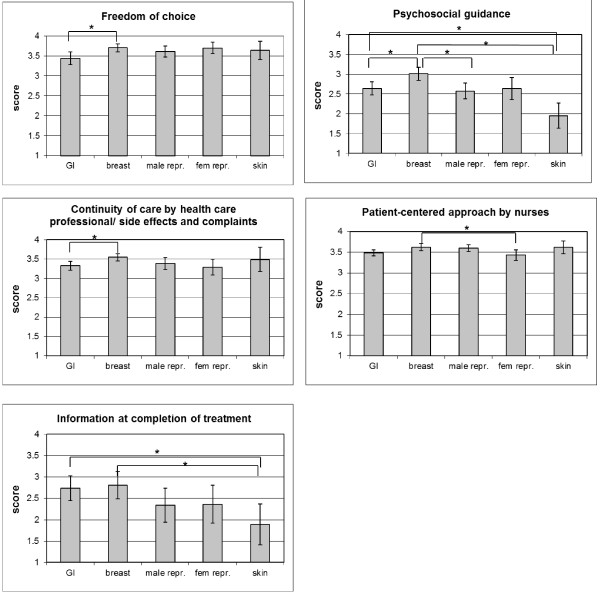
**Average scale score and 95% confidence intervals for scales with statistically significant differences in average score between tumour types (ANOVA p<0.05).** Differences in average scale scores are reported for the five most commonly reported tumour types. Calculations are based on 205 tumours of the digestive tract, 171 breast tumours, 153 tumours of the male reproductive organs, 73 tumours of the female reproductive organs and 70 tumours of the skin.

Average scale scores based on the experience questionnaire can be found in Figure 1. On average hospitals had low scores for ‘Psychosocial guidance’, ‘Other investigations and treatments’ and ‘Information at completion of treatment’. Highest scores were found for ‘Skills and cooperation of healthcare professionals’ and for ‘Patient-centered approach by doctors’. Doctors scored significantly higher than nurses with regard to patient-centered approach (p<0.001). The average scale scores differed significantly (p<0.05) depending on the tumour type for five scales (Freedom of choice, Psychosocial guidance, Continuity of care by healthcare professional/side effects and complaints, Patient-centered approach by nurses and Information at completion of treatment) (see Figure 2). Highest scores were invariably given by breast cancer patients. The lowest scores were given by patients with gastro-intestinal cancer, skin cancer and/or cancer of the female reproductive organs, depending on the scale.

### Improvement scores

We used the experience and importance questionnaires to determine improvement scores in order to identify aspects where patients had a high percentage of bad experiences combined with a high average importance score. Our analyses showed that the aspect with the highest improvement score was ‘if your cancer is heritable, was examination of your relatives discussed?’. Up to 70 percent of patients, for whom this was relevant (i.e. they did not indicate the question was not applicable), reported a negative experience with this aspect. The ten most important aspects included two questions regarding heritable cancer, four questions regarding aftercare and two questions regarding planning of appointments (see Table 5).

**Table 5:** Improvement scores for, ten questions with the highest improvement score and ten questions with the lowest improvement score

**Question**	**Average importance score**	**% negative experiences**	**Improvement score**
**Ten highest improvement scores**			
If your cancer is heritable, was examination of your relatives discussed?	3.35	69.34	2.33
After completion of the treatment, were you called at home within a week by a healthcare professional to discuss how you were doing?	2.81	71.71	2.01
Did you receive information regarding participation in medical research?	2.76	72.10	1.99
After completion of the treatment, did you receive information regarding the possibilities of care for your emotions?	2.86	66.18	1.89
Were you informed if the waiting time was longer than expected	2.80	63.30	1.77
Were multiple appointments in this hospital planned for the same day?	3.25	53.21	1.73
During aftercare, were you offered help with problems surrounding your feelings?	2.86	57.32	1.64
Did healthcare professionals inform you about patient organisations?	2.53	63.35	1.61
During aftercare, were you offered help with questions regarding getting back into your normal daily routine?	2.76	57.75	1.59
If your cancer is heritable, are you regularly checked for any new tumours?	3.63	41.90	1.52
**Ten lowest improvement scores**			
Were the doctors polite to you?	3.08	1.93	0.06
Were the nurses polite to you?	2.98	2.21	0.07
Were doctors and nurses aware of your situation?	3.60	2.06	0.07
Did the doctors and nurses treat you in a skilful manner?	3.44	2.6	0.09
Was written information clear?	3.20	2.87	0.09
Did healthcare professionals give you contradictory information?	3.50	2.63	0.09
Did the information you received beforehand correspond well with what happened during investigation and treatment?	3.33	3.54	0.12
Did the doctors take you seriously?	3.49	3.45	0.12
Did the doctor give you the test results personally?	3.40	3.57	0.12
Did you trust the doctors and nurses?	3.63	3.46	0.13

For each question the average importance score is shown as measured in the importance questionnaire (1 is lowest, 4 is highest), the percentage of negative experiences (the two most negative categories were taken together, for instance ‘none’ and ‘some’ or ‘never’ and sometimes’).

The lowest improvement scores regarded attitude and information provision by healthcare professionals. The low scores were caused mainly by the low percentage of negative experiences (1.93-3.46 percent) reported (see Table 5).

## Discussion

We developed a questionnaire that measures patient experiences with cancer care in hospitals for patients with all types of cancer. The revised version of the questionnaire contains 99 questions and 12 reliable scales (the original questionnaire prior to psychometric analyses contained 110 questions). It measures a broad array of topics specific to the needs and wishes of cancer patients, including provision of information at different stages of the treatment, continuity of care, psychosocial guidance and personal attention after completion of the treatment. For the majority of scales and for the items rated most important by patients we found no differences between tumour types, although certain tumour-specific results were found. This illustrates the advantage of a generic questionnaire. It can be used for patients with all tumour types (compared to having to develop many tumour-specific questionnaires). Moreover, a generic questionnaire can be sent out to many patients per hospital, thereby making it possible to make large scale comparisons between hospitals.

With regard to our first research question - What are the most important aspects of quality of cancer care according to patients? - we found that the most important themes were: rapid and adequate referral, consultation of other professionals by the doctor, rapid start of the treatment after diagnosis, the need for information and confidence in the healthcare professional (Table 3). The first three were previously identified in breast cancer patients [20,30]. The latter two partially confirm previous findings (provision of information, communication skills, accessibility of care and technical quality of the medical care) [17-20]. Important items from the theory of patient-centeredness can also be identified in these themes (collaboration and team management, education and shared knowledge, free flow and accessibility of information), supporting the construct validity of these subscales.

The answer to our second research question - What are the actual experiences of cancer patients with these quality aspects? can be derived from the scores on the different scales in the experience questionnaire (Figure 1). Highest scores were given for skills and cooperation of healthcare professionals and for patient-centered approach by doctors. Interestingly, hospitals scored relatively low on psychosocial guidance, on possibilities for other investigations and treatments and on information after completion of the treatment. In addition, our improvement scores showed that examination of relatives of patients with a heritable type of cancer was not frequently discussed with patients even though patients found this to be important. A study published in 2009 by Damman et al. among breast cancer patients reported high improvement scores for referral to rehabilitation, the possibility of making an appointment rapidly and referral to psychosocial care [10]. This is generally in agreement with our findings.

Our third research question was ‘Are there any differences in experiences between subgroups of cancer patients?’ Based on the experience questionnaire, we observed statistically significant differences between patients with different tumour types for five scales (Freedom of choice, Psychosocial guidance, Continuity of care by healthcare professional/side effects and complaints, Patient-centered approach by nurses and Information at completion of treatment). Breast cancer patients reported most positive experiences for all these scales (Figure 2). This can be explained by the improvement in the quality and speed of breast cancer detection and treatment in the Netherlands in the recent years [31]. In contrast to most studies, our questionnaire allows participants to rate doctors and nurses separately. This resulted in significantly higher scores for doctors compared to nurses regarding their attitude towards patients. This is a surprising result because in other studies the contrary has been found [32,33]. Moreover, from the focus groups we had the impression that nurses are found to be more patient-centred than physicians. It must be noted that the low number of respondents with cancer of the urinary tract, head and neck, and central nervous system, prevent us from drawing conclusions regarding the use of the questionnaire in specific groups of patients.

A major strength of this study is that we incorporated the importance cancer patients attach to different quality aspects in several phases of the construction of the questionnaire. Our focus group discussions with cancer patients ensured that quality aspects important to patients were included in the pilot version of the questionnaire. Sending out the importance questionnaire enabled us to exclude questions that are considered unimportant by patients and to retain questions that would be eliminated from the questionnaire based on their psychometric properties but were deemed important by patients. Our questionnaire includes a broad range of themes, derived from multiple sources and is therefore very comprehensive. An additional advantage is that the questionnaire is generic for cancer patients. This allows for the comparison of different tumour types. Moreover, our results allow policy makers, healthcare professionals and patient organisations to learn from the type of care given to breast cancer patients, as we show high scores are given by breast cancer patients.

A possible drawback of the comprehensive nature of the questionnaire is that it may be considered long by patients, although only 13 (out of 732) participants reported this complaint. The experience questionnaire showed a response of 50 percent. This is comparable to what is found in the literature (42–75 percent) [34-38]. The literature is not in agreement as to whether the number of questions in a questionnaire influences the response rate. Sitzia et al. found in 1998 in a review among 210 studies that there was no effect, while Edwards et al. found in a review in 2002 that shorter questionnaires have higher response rates [36,39,40]. It appears as though the number of questions in the questionnaire, although rather high, did not form a substantial barrier for patients in the current study.

There was a difference in response between the experience questionnaire (50 percent) and the importance questionnaire (68 percent). It is unclear how this difference arose, since the distribution of age and gender for respondents and non-respondents in both groups was highly similar, the number of questions was comparable in both questionnaires and invitations to participate in the study were sent in the same way for both questionnaires. The only substantial difference found between the two groups was in the types of tumours reported by the patients. This may have caused a different response. Compared to the Dutch cancer population, there were fewer patients with lower respiratory tract cancer and more with cancer of the female reproductive organs among respondents to the experience questionnaire. It remains unclear why this difference exists since respondents from the different diagnosis groups were all selected and approached in exactly the same way. The respondents to the importance questionnaire had the same five most frequently reported tumour types as the Dutch cancer population, making systematic sampling errors unlikely.

The fact that the questionnaire is in Dutch also introduces a possible bias. Dutch residents who do not speak the Dutch language are less likely to fill out the questionnaire, even though we allowed people to fill out the questionnaire with the aid of an interpreter. This was reflected in our results, 94 percent of the respondents were of Dutch descent. This is observed as well in other questionnaire-based studies, 70–79 percent of a study population in New Zealand and the USA was Caucasian [41,42]. The translated version of the questionnaire (additional file 1) will enable international comparisons.

In order to keep the questionnaire down to a reasonable size we did not include certain aspects of cancer care that only apply to specific subgroups. This may be considered a limitation. However, CQI questionnaires for specific subgroups have been or are being developed for breast cancer care [10] and for radiotherapy [43]. The combination of these questionnaires will result in both complete and specific information on the experiences of the cancer population.

Although the sample for this study was drawn by a Dutch health insurance company with national coverage, no data are available on the hospitals where the respondents were treated. Therefore we cannot guarantee national coverage of our data. In future research stratification on hospital level should be applied so that national coverage of the respondent population can be ensured and results can be generalized to the whole country.

Based on the research described here, the current questionnaire should be tested for its ability to discriminate between hospitals, so it can be used for comparison of hospitals. Preferably this is to be done in a group of cancer patients with one specific tumour type, to rule out any potential differences caused by the tumour type. Furthermore, using the English translated version of the questionnaire will facilitate international use of the questionnaire and comparison with international cancer care.

## Conclusion

We developed a questionnaire that measures the importance of aspects and the experiences of all types of cancer patients based on quality aspects formulated by patients. We showed known and new quality aspects that are very important to patients, the experiences of patients with respect to these aspects, and that differences exist in experiences between people with different tumour types. After testing for discriminatory power our questionnaire can be used nation-wide to measure quality of cancer care from the patient perspective and to identify differences in the experiences of patients in different hospitals. Finally, in contrast to most previously developed questionnaires, our questionnaire makes a distinction between care provided by doctors and by nurses.

## Competing interests

The author(s) declare that they have no competing interests.

## Author’s contributions

JB analyzed and interpreted the data and drafted the manuscript, MZ contributed to the conception and design of the study, collected data and critically read the manuscript. PE contributed to the conception and design of the study and critically read the manuscript, MH contributed to the conception and design of the study and critically read the manuscript, DD contributed to the conception and design of the study and critically read the manuscript, JR contributed to the conception and design of the study and critically read the manuscript. All authors read and approved the final manuscript.

## Pre-publication history

The pre-publication history for this paper can be accessed here:

http://www.biomedcentral.com/1471-2407/13/203/prepub

## References

[B1] Tishelman C Lovgren M Broberger E Hamberg K Sprangers MA Are the most distressing concerns of patients with inoperable lung cancer adequately assessed? a mixed-methods analysis J Clin Oncol 2010 28 1942 1949 10.1200/JCO.2009.23.3403 20212257

[B2] Institute of Medicine Crossing the quality chasm: a New health system for the 21st century 2001 Washington, DC: National Academy Press 25057539

[B3] Hahn SR Patient-centered communication to assess and enhance patient adherence to glaucoma medication Ophthalmology 2009 116 S37 S42 10.1016/j.ophtha.2009.06.023 19837259

[B4] Meterko M Wright S Lin H Lowy E Cleary PD Mortality among patients with acute myocardial infarction: the influences of patient-centered care and evidence-based medicine Health Serv Res 2010 45 1188 1204 10.1111/j.1475-6773.2010.01138.x 20662947 PMC2965500

[B5] Swenson SL Buell S Zettler P White M Ruston DC Lo B Patient-centered communication: do patients really prefer it? J Gen Intern Med 2004 19 1069 1079 10.1111/j.1525-1497.2004.30384.x 15566435 PMC1494788

[B6] Groene O Patient centredness and quality improvement efforts in hospitals: rationale, measurement, implementation Int J Qual Health Care 2011 23 531 537 10.1093/intqhc/mzr058 21862449

[B7] Lilford R Mohammed MA Spiegelhalter D Thomson R Use and misuse of process and outcome data in managing performance of acute medical care: avoiding institutional stigma Lancet 2004 363 1147 1154 10.1016/S0140-6736(04)15901-1 15064036

[B8] Wessels H de Graeff A Wynia K de Heus M Kruitwagen CL Teunissen SC Voest EE Are health care professionals able to judge cancer patients' health care preferences correctly? A cross-sectional study. BMC Health Serv Res 2010 10 198 10.1186/1472-6963-10-198 PMC2911431 20615226

[B9] Cronin C Patient-centered care: an overview of definitions and concepts 2004 Washington, DC: Prepared for the National Health Council

[B10] Damman OC Hendriks M Sixma HJ Towards more patient centred healthcare: a new consumer quality index instrument to assess patients' experiences with breast care Eur J Cancer 2009 45 1569 1577 10.1016/j.ejca.2008.12.011 19167212

[B11] Delnoij D Rademakers J Groenewegen PP The dutch consumer quality index: an example of stakeholder involvement in indicator development BMC Health Serv Res 2010 10 88 10.1186/1472-6963-10-88 20370925 PMC2864255

[B12] Zuidgeest M Measuring and improving the quality of care from the healthcare user perspective:the consumer quality index 2011 Bergenschenhoek, Netherlands: PhD Thesis

[B13] Fung CH Lim YW Mattke S Damberg C Shekelle PG Systematic review: the evidence that publishing patient care performance data improves quality of care Ann Intern Med 2008 148 111 123 10.7326/0003-4819-148-2-200801150-00006 18195336

[B14] Hendriks M Spreeuwenberg P Rademakers J Delnoij DM Dutch healthcare reform: did it result in performance improvement of health plans? A comparison of consumer experiences over time. BMC Health Serv Res 2009 9 167 10.1186/1472-6963-9-167 PMC2761896 19761580

[B15] Hargraves JL Hays RD Cleary PD Psychometric properties of the consumer assessment of health plans study (CAHPS) 2.0 Adult core survey Health Serv Res 2003 38 1509 1527 10.1111/j.1475-6773.2003.00190.x 14727785 PMC1360961

[B16] Sixma HJ Kerssens JJ Campen CV Peters L Quality of care from the patients' perspective: from theoretical concept to a new measuring instrument Health Expect 1998 1 82 95 10.1046/j.1369-6513.1998.00004.x 11281863 PMC5139902

[B17] Wiggers JH Donovan KO Redman S Sanson-Fisher RW Cancer patient satisfaction with care Cancer 1990 66 610 616 10.1002/1097-0142(19900801)66:3<610::AID-CNCR2820660335>3.0.CO;2-T 2364373

[B18] Skarstein J Dahl AA Laading J Fossa SD ‘Patient satisfaction’ in hospitalized cancer patients Acta Oncol 2002 41 639 645 10.1080/028418602321028256 14651208

[B19] Tamburini M Gangeri L Brunelli C Boeri P Borreani C Bosisio M Karmann CF Greco M Miccinesi G Murru L Cancer patients’ needs during hospitalisation: a quantitative and qualitative study BMC Cancer 2003 3 12 10.1186/1471-2407-3-12 12710890 PMC155542

[B20] Wessels H de Graeff A Wynia K Sixma HJ de Heus M Schipper M Woltjer GT Teunissen SC Voest EE Medical oncology patients' preferences with regard to health care: development of a patient-driven questionnaire Ann Oncol 2009 20 1708 1713 10.1093/annonc/mdp044 19497943

[B21] Cleary PD Edgman-Levitan S Health care quality Incorporating consumer perspectives. JAMA 1997 278 1608 1612 9370508

[B22] NFK quality criteria 2010 http://www.nfk.nl/publicaties/kwaliteitscriteria/_pid/content1/_rp_content1_elementId/1_149788

[B23] NPCF quality criteria 2010 http://www.npcf.nl/index.php?option=com_virtuemart&page=shop.product_details&flypage=flypage.tpl&category_id=1&product_id=2&Itemid=75&vmcchk=1&Itemid=75

[B24] EORTC IN PATSAT32 2010 http://groups.eortc.be/qol/questionnaires_eortcinpatsat32.htm

[B25] Sixma H Delnoij D Measuring patient experiences in the netherlands: the case of hospital care Eur J Pub Health 2008 18 88

[B26] Dillman DA Mail and telephone surveys: the total design method 1978 New York: John Wiley & Sons

[B27] Sixma HJ Boer De D Delnoij D Handboek CQ-index ontwikkeling: richtlijnen en voorschriften voor de ontwikkeling van een CQ-index meetinstrument 2008 Utrecht: NIVEL

[B28] Dutch National cancer registration website Dutch national cancer registration website 2012 http://www.cijfersoverkanker.nl/

[B29] WHO Process of translation and adaptation of instruments 2013 http://www.who.int/substance_abuse/research_tools/translation/en/

[B30] de Boer D Delnoij D Rademakers J Do patient experiences on priority aspects of health care predict their global rating of quality of care? a study in five patient groups Health Expect 2010 13 285 297 20550597 10.1111/j.1369-7625.2010.00591.x PMC5060537

[B31] Henselmans I Sanderman R Smink A Ranchor AV De Vries J Waiting times in breast disease clinics and psychological well-being: speedy care is better care Ned Tijdschr Geneeskd 2010 154 B491 20170572

[B32] Wilson IB Landon BE Hirschhorn LR McInnes K Ding L Marsden PV Cleary PD Quality of HIV care provided by nurse practitioners, physician assistants, and physicians Ann Intern Med 2005 143 729 736 10.7326/0003-4819-143-10-200511150-00010 16287794

[B33] Roblin DW Becker ER Adams EK Howard DH Roberts MH Patient satisfaction with primary care: does type of practitioner matter? Med Care 2004 42 579 590 10.1097/01.mlr.0000128005.27364.72 15167326

[B34] Ikkersheim DE Koolman X Dutch healthcare reform: did it result in better patient experiences in hospitals? A comparison of the consumer quality index over time. BMC Health Serv Res 2012 12 76 10.1186/1472-6963-12-76 PMC3326705 22443174

[B35] de Boer D Delnoij D Rademakers J The discriminative power of patient experience surveys BMC Health Serv Res 2011 11 332 10.1186/1472-6963-11-332 22145965 PMC3292538

[B36] Sitzia J Wood N Response rate in patient satisfaction research: an analysis of 210 published studies Int J Qual Health Care 1998 10 311 317 10.1093/intqhc/10.4.311 9835247

[B37] Sanson-Fisher R Girgis A Boyes A Bonevski B Burton L Cook P The unmet supportive care needs of patients with cancer. Supportive care review group Cancer 2000 88 226 237 10.1002/(SICI)1097-0142(20000101)88:1<226::AID-CNCR30>3.0.CO;2-P 10618627

[B38] Ross L Petersen MA Johnsen AT Lundstrom LH Groenvold M Are different groups of cancer patients offered rehabilitation to the same extent? a report from the population-based study "the cancer Patient's world" Support Care Cancer 2012 20 1089 1100 10.1007/s00520-011-1189-6 21597939

[B39] Zuidgeest M De Boer D Hendriks M Rademakers J Verschillende dataverzamelingsmethoden in CQI onderzoek: een overzicht van de respons en representativiteit van respondenten Tijdschrift voor gezondheidswetenschappen 2008 86 8 455 462 10.1007/BF03082143

[B40] Edwards P Roberts I Clarke M DiGuiseppi C Pratap S Wentz R Kwan I Increasing response rates to postal questionnaires: systematic review BMJ 2002 324 1183 10.1136/bmj.324.7347.1183 12016181 PMC111107

[B41] Whitehead L Methodological issues in internet-mediated research: a randomized comparison of internet versus mailed questionnaires J Med Internet Res 2011 13 e109 10.2196/jmir.1593 22155721 PMC3278095

[B42] Ritter P Lorig K Laurent D Matthews K Internet versus mailed questionnaires: a randomized comparison J Med Internet Res 2004 6 e29 10.2196/jmir.6.3.e29 15471755 PMC1550608

[B43] Nijman JL Sixma H Van TB Keus RB Hendriks M The quality of radiation care: the results of focus group interviews and concept mapping to explore the patient's perspective Radiother Oncol 2012 102 154 160 10.1016/j.radonc.2011.08.005 21907440

